# Harvard University

**Published:** 1847-05

**Authors:** 


### HARVARD UNIVERSITY.

We announced in our last number the resignation by Professor John C. Warren of the Chair of Anatomy and Surgery in Harvard University. The board of trustees have paid him the compliment, richly merited, of electing him *Emeritus* Professor of Anatomy and

Surgery. We learn from the Boston Medical and Surgical Journal that they have appointed three new professors since the resignation of Dr. Warren, two of whom are to be attached to the Medical College in Boston, and one to the University of Cambridge. The new incumbents are—Oliver W. Holmes, M.D., Professor of Anatomy and Physiology; John B. S. Jackson, M.D., Professor of Pathological Anatomy, and Curator; and Jeffries Wyman, M.D., Hersey Professor of Anatomy at Cambridge. These gentlemen enjoy a high reputation for talents, industry, and learning.

